# Sex- and Age-Dependent Wide-Field Choroidal Thickness Differences in Healthy Eyes

**DOI:** 10.3390/jcm12041505

**Published:** 2023-02-14

**Authors:** Naohisa Mihara, Shozo Sonoda, Hiroto Terasaki, Hideki Shiihara, Takato Sakono, Ryoh Funatsu, Taiji Sakamoto

**Affiliations:** Department of Ophthalmology, Kagoshima University Graduate School of Medical and Dental Sciences, 8-35-1 Sakuragaoka, Kagoshima 890-8544, Japan

**Keywords:** choroidal thickness, normal eyes, posterior pole, optic disc, optical coherence tomography, vortex vein, watershed

## Abstract

In this study, we aimed to map and characterize the choroidal thickness over a wide area from the posterior pole to the vortex vein in normal eyes. This observational study included 146 healthy eyes (63 male). Three-dimensional volume data were acquired to create a choroidal thickness map using swept-source optical coherence tomography. The map was classified as type A if an area with a choroidal thickness >250 µm in the vertical direction from the optic disc, and the area corresponding to the watershed was not observed, or as type B if such an area was observed. The relationship between the ratio of groups A to B and age was compared by classifying the age for three age groups: <40, 40–60, and >60 years in men and women. In men and women, 69.8% and 49.4% were classified as type A, respectively, with significant sex differences (*p* = 0.013). The proportion of type B decreased with increasing age in both the sexes. There was a significant difference between ≤60 and >60 years in men and between ≤40 and >40 years in women (*p* < 0.05). To conclude, the wide-area choroidal thickness and the age-dependent changes in healthy eyes differed between the sexes.

## 1. Introduction

The human choroid contains a tremendous amount of blood-rich tissue and plays an important role in maintaining retinal function [1]. Changes in the choroidal structure in diseased eyes have been previously reported, suggesting the involvement of the choroid in their pathogenesis. Recently, the concept of pachychoroid spectrum disorders (PSDs) has been proposed as a better description of the pathogenesis of CSC and polypoidal choroidal vasculopathy (PCV) [2].

PSDs are often associated with vascular abnormalities in the choroidal Haller layer, including anomalous anastomosis or asymmetry of the Haller vessels [3,4,5]. The occurrence is believed to be owing to stasis of the choroidal Haller vessels, which is explained by the concept of venous overload choroidopathy [4]. In general, blood stasis causes dilation of the blood vessels, and stasis of the choroidal circulation results in the thickening of the choroid [6].

Indocyanine green angiography (ICGA) images of PSD eyes have demonstrated that the peripheral choroid, including the vortex veins, is involved in the pathogenesis of the disease [7,8], and the analysis of the peripheral choroid has attracted attention. However, ICGA images present two-dimensional data and are limited to the analysis of the position and running pattern of blood vessels. Furthermore, information on choroidal thickness, an important factor in the analysis of the choroid, which is essentially a three-dimensional (3D) structure, is unavailable. Considering the importance of venous remodeling in the pathogenesis of various choroid-related diseases and venous overload choroidopathy, understanding the status and changes in choroidal thickening in both the posterior pole and the entire choroid is important.

Wide-field (WF) swept-source optical coherence tomography (SS-OCT) has recently made the measurement of choroidal thickness possible in a range that includes the vortex vein ampullae. Several studies have been reported on CSC [9] and myopic eyes [10,11] using wide-angle OCT, which has provided new insights. However, it has not yet been reported on normal eyes. In this study, we aimed to obtain WF-OCT images and used them to map and characterize the choroidal thickness over a wide area from the posterior pole to the vortex vein in normal eyes.

## 2. Materials and Methods

### 2.1. Study Design

This single-center, retrospective, observational study was approved by the Kagoshima University Ethics Committee (no. 160121, on 3 August 2021). All the procedures were performed in accordance with the guidelines of the Declaration of Helsinki.

### 2.2. Study Population

The healthy eyes of Japanese patients who visited the Ophthalmology Department of Kagoshima University Hospital between April 2021 and 2022 and those who requested an examination, including OCT, at the time of their eye examinations were included in this study. The refraction (RM8900, Topcon Corp., Tokyo, Japan), corrected visual acuity, which was measured with the Landolt C chart, ocular axis length (Tomey GmbH, Nuremberg, Germany), and WF-OCT images (Xephilio OCT-S1; Canon Medical Systems Corp., Tochigi, Japan) were measured.

Patients with an ocular axis length <20 mm and >27 mm, equivalent sphere <−6D and >+3D, unclear WF-OCT images (signal strength less than or equal to 5), history of buckle surgery or vitrectomy, or examination results that precisely indicated uveitis, choroidal retinal disease, glaucoma, posterior uveitis, pregnancy, or neoplastic disease were excluded.

### 2.3. Evaluation of Choroidal Thickness by WF-OCT

The fundus was imaged using WF-OCT with 1010–1110 nm near-infrared illumination (scanning laser ophthalmoscope, 780 nm) at a scanning speed of 100,000 A-scans/s. SS-OCT was used to acquire 3D volume data of 20 mm (128 B scans; length) × 23 mm (1024 pixels; width) × 5.3 mm (1396 pixels; depth) for measuring the choroidal thickness. To avoid the effect of diurnal variation, the choroidal thickness was measured from 2:00 to 4:00 p.m. The vertical distance from the Bruch membrane to the scleral border was used as the choroidal thickness for choroid segmentation.

Segmentation was performed automatically using built-in software (Xephilio OCT-S1; Canon Medical Systems Corp.) supported by artificial intelligence. Two examiners (NM and RF) evaluated the automatic segmentation in all the slices and manually modified the segmentation; when one evaluator deemed the segmentation inappropriate, the other evaluator was consulted for confirmation. The segmentation was then modified if considered inappropriate.

### 2.4. Classification of the Choroidal Map Patterns

**Figure 1:**
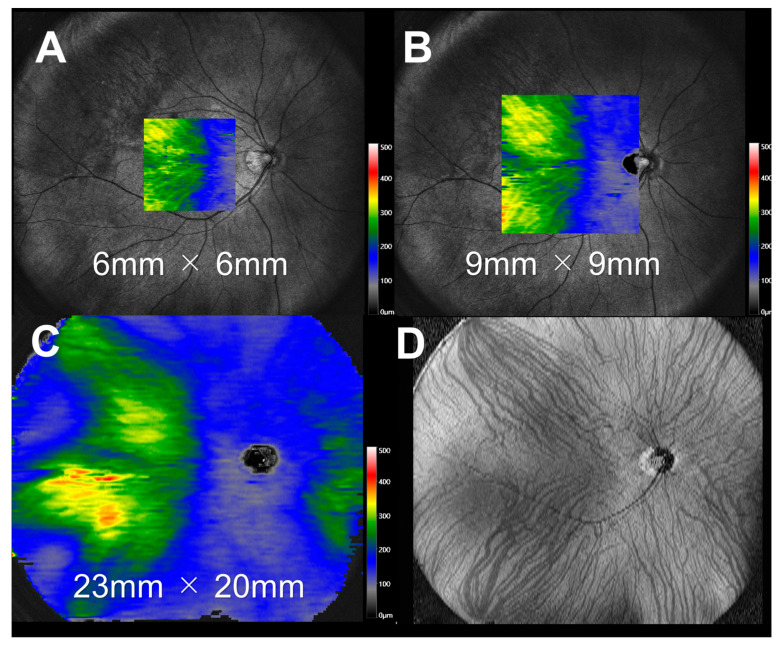
Choroidal thickness map. (**A**) Choroidal thickness map of 6 × 6 mm^2^. (**B**) Choroidal thickness map of 9 × 9 mm^2^. (**C**) Choroidal thickness map of 23 × 20 mm^2^ (generated from 128 B-scan images). (**D**) Choroidal en face image generated from the same images.

Former studies [12,13] have analyzed scan widths of 6 mm (Figure 1A) or 9 mm (Figure 1B). However, in this study, we created a choroidal thickness map based on the B-scan images (Figure 1C). The en face image (Figure 1D), which was created from the same image, shows that the analysis covers the area from the posterior pole of the fundus to the vortex vein, which is a wider area than that in the previous analyses.

The choroid is functionally divided into four independent quadrants by vertical and horizontal watersheds based on the drainage route [14]. Our previous report [15] revealed that the choroidal thickness above the optic disc varied widely, whereas the choroidal thickness below the optic disc showed less variation in most participants. Thus, we attempted to formulate a classification based on the choroidal thickness above the optic disc. We used 250 µm as the standard for the classification based on a report that the average choroidal thickness in the upper quadrant to the optic disc at 2000 μm distal is 229 µm [16] Hayreh et al. [14] reported that there are many variations in the choroidal watershed. However, most are located in the vertical region centered on the optic disc. We distinguished a 3-mm-wide region centered on the optic disc as the watershed and evaluated choroidal thickness in the choroidal watershed.

**Figure 2:**
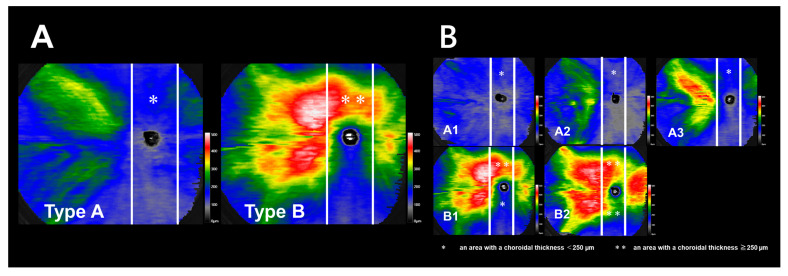
Classification of the choroidal thickness map. (**A**) Two white lines were placed to mark a 3-mm-wide area in the center of the disc. The choroidal thickness map is classified as type A if an area with a choroidal thickness ≥250 µm in the vertical direction from the optic disc in the area corresponding to the watershed is not observed or as type B if such an area is observed. (**B**) Type A is divided into three subgroups: type A1, those with an overall thickness of less than 250 µm; type A2, those with an area ≥250 µm on the temporal side only; type A3, those with an area ≥250 µm on both the temporal and nasal sides. Type B is subdivided into two types: B1, those with an area ≥250 µm only above the disc; B2, those with an area ≥250 µm below the disc. * and ** indicate an area with a choroidal thickness <250 um and ≥250 um.

The wide-angle choroidal map was classified as type A if an area larger than 250 µm in a vertical region (approximately 3 mm wide centered on the optic disc and connecting the adjacent areas on the nasal and temporal sides) was not observed; however, if such an area was present, it was classified as type B (Figure 2A).

Type A was further divided into three subgroups: A1 was defined as the absence of an area ≥250 µm in the choroidal thickness map, A2 as an area ≥250 µm on the temporal side only, and A3 as an area ≥250 µm on both the temporal and nasal sides. Type B was divided into two subgroups: B1 was defined as areas ≥250 µm only above the optic disc, and B2 as areas ≥250 µm below the optic disc (Figure 2B).

### 2.5. Verification of Reproducibility of Choroidal Map Image Classification

Choroidal map images were classified by two raters blinded to the study based on the abovementioned method. Pattern agreement for each image was assessed by calculating simple and weighted kappa (κ) statistics. Linear weights were used to calculate weighted κ values, and κ statistics were interpreted using the ranges proposed by Landis and Koch [17] (<0, poor agreement; 0–0.20, slight agreement; 0.21–0.40, normal agreement; 0.41–0.60, moderate agreement; 0.61–0.8, substantial agreement; >0.81, near-perfect agreement).

### 2.6. Statistical Analysis

All the statistical analyses were performed using a commercial statistical package (SPSS Statistics version 28; IBM Corp., Armonk, NY, USA). The chi-squared test was used for categorical variables, and the Mann–Whitney U test was used for nonparametric continuous variables. Bivariate correlations were assessed using Spearman’s correlation coefficient. The stepwise regression method of multiple regression was used. The Kruskal–Wallis test was used for comparisons between multiple groups, and multiple tests were compared for each group using the Bonferroni method. A *p*-value < 0.05 was considered statistically significant.

## 3. Results

### 3.1. Background Characteristics of Healthy Eyes

The background characteristics of the participants are presented in Table 1. Only the axial length (*p* = 0.02) differed significantly between the male and female participants, as opposed to the age, central choroidal thickness (CCT), and SE, which did not differ significantly.

### 3.2. Relationship of CCT to SE, Ocular Axis Length, and Age

**Figure 3:**
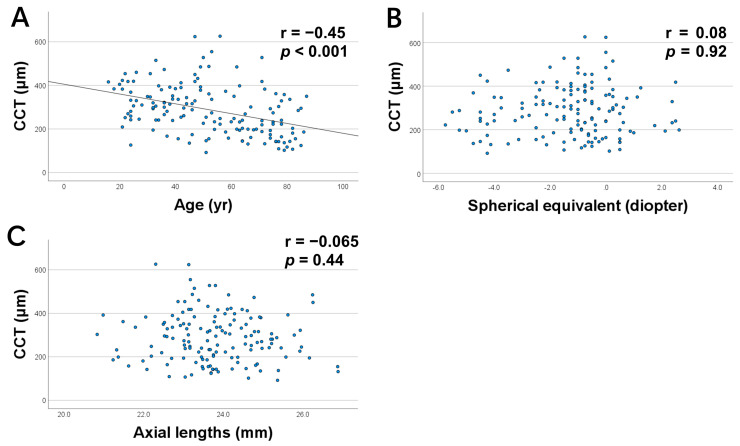
Scatter plots of central choroidal thickness (CCT) versus age, axial length, and spherical equivalent. CCT correlated with (**A**) age alone but not with (**B**) axial length or (**C**) spherical equivalent.

Scatter plots of the CCT and age, ocular axis, and SE are presented in Figure 3. Only the age correlated with the CCT (r = −0.45, *p* < 0.001; Figure 3A), SE (r = 0.08, *p* = 0.92; Figure 3B), and axial length (r = −0.065, *p* = 0.44; Figure 3C).

The multiple regression analysis on CCT, which was performed using the age, ocular axial length, and SE as explanatory variables, revealed that the age and axial length correlated with the CCT (β = −0.52, *p* < 0.001; β = −0.29, *p* < 0.001; Table 2).

Age and axial lengths were selected as markers of central choroidal thickness in a stepwise regression analysis, and the spherical equivalent was excluded.

### 3.3. Classification of the Context Map Patterns

**Figure 4:**
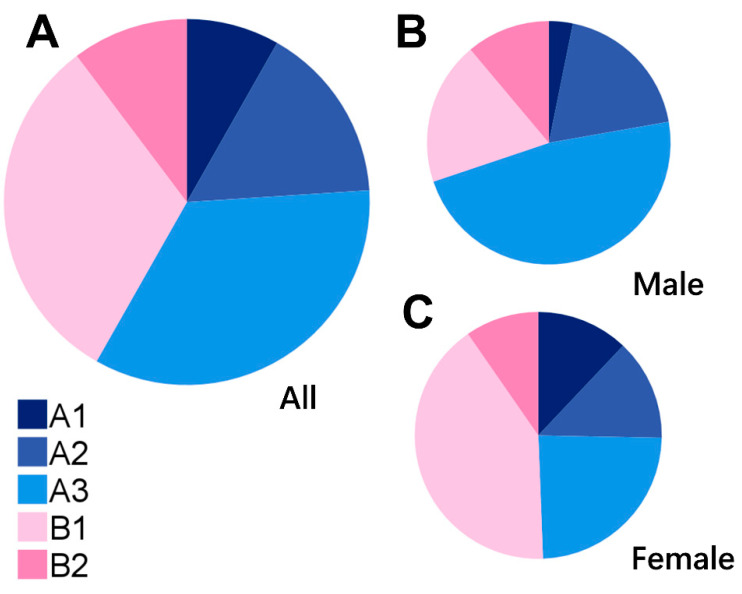
Pie chart of the choroidal map pattern classification. (**A**) Overall: A1, 12 (8.2%); A2, 23 (15.8%); A3, 50 (34.2%); B1, 46 (31.5%); B2, 15 (10.3%). (**B**) Men: A1, 2 (3.2%); A2, 12 (19.0%); A3, 30 (47.6%); B1, 12 (19.1%); B2, 7 (11.1%). (**C**) Women: A1, 10 (12.0%); A2, 11 (13.3%); A3, 20 (24.1%); B1, 34 (41.0%); B2, 8 (9.6%).

The agreement in image classification between the two raters (NM and TS) was nearly perfect (unweighted κ = 0.94, linearly weighted κ = 0.96). Figure 4 presents a pie chart of the classification of choroidal map pattern percentages in all the participants (Figure 4A), males (Figure 4B) and females (Figure 4C). Type A3 was more common in all the cases, followed by Types B1 and A2. These three types constituted more than 80% of all the cases (Figure 4A). The chi-squared test revealed a significant difference of the proportions of the five patterns between men and women (*p* < 0.01, Figure 4B,C).

### 3.4. Relationship between the Choroidal Map Patterns and Biomarkers

**Figure 5:**
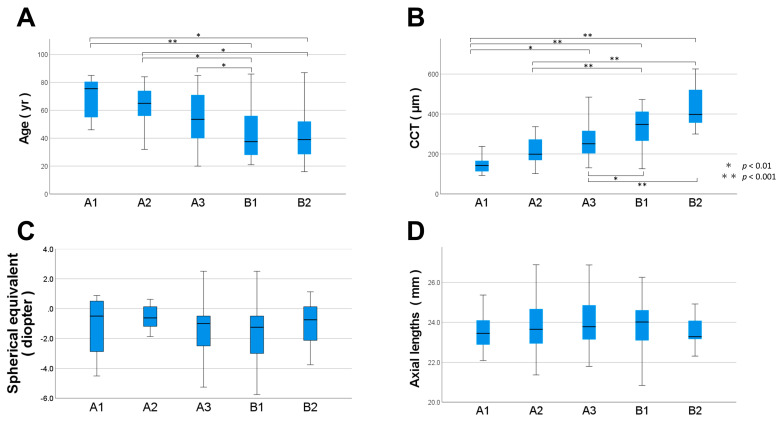
Box-and-whisker plot of (**A**) age, (**B**) central choroidal thickness, (CCT), (**C**) axial length, and (**D**) spherical equivalent for each of the five classification groups. Age and central choroidal thickness significantly varied among the five groups.

Box-and-whisker plots of age, CCT, axial length, and SE for each of the five groups overall are shown in Figure 5. The mean age was 69.5 ± 13.9, 61.7 ± 16.0, 55.0 ± 18.9, 43.6 ± 18.5, and 41.2 ± 18.6 years for A1, A2, A3, B1, and B2, respectively (Figure 5A). The Kruskal–Wallis test showed a significant difference in the age (*p* < 0.05) among the five patterns, with comparisons between each pattern showing significant differences between A1 and B1 (*p* < 0.001), A1 and B2 (*p* = 0.003), A2 and B1 (*p* = 0.003), A2 and B2 (*p* = 0.024), and A3 and B1 (*p* = 0.024).

The mean CCT values were 145.3 ± 41.3, 218.4 ± 66.7, 265.5 ± 92.3, 334.4 ± 88.6, and 440.7 ± 106.7 µm for A1, A2, A3, B1, and B2, respectively (Figure 5B). The results of the Kruskal–Wallis test for CCT showed significant differences among the five patterns (*p* < 0.05), and comparisons between the patterns showed significant differences between A1 and A3 (*p* = 0.003), A1 and B1 (*p* < 0.001), A1 and B2 (*p* < 0.001), A2 and B1 (*p* < 0001), A2 and B2 (*p* < 0.001), A3 and B1 (*p* = 0.008), and A3 and B2 (*p* < 0.001).

The mean SE values were −1.17 ± 2.02, −0.67 ± 1.38, −1.43 ± 1.87, −1.62 ± 1.98, and −1.01 ± 1.36 D for A1, A2, A3, B1, and B2, respectively (Figure 5C). The mean axial lengths were 23.52 ± 0.93, 23.73 ± 1.33, 23.97 ± 1.15, 23.78 ± 1.28, and 23.66 ± 0.92 mm for A1, A2, A3, B1, and B2, respectively (Figure 5D). The Kruskal–Wallis test results for the SE and axial length showed no significant difference among the five groups.

### 3.5. Differences in the Choroidal Thickness Map Patterns by Sex and Age Group

The above results revealed a relationship between the map patterns and sex, age, and CCT. Therefore, we divided the patients into three groups according to age: young (<40 years), middle (40–60 years), and old (≥60 years), and compared the ratios of types A and B for all the participants and each sex. The young group had 47 subjects (20 males and 27 females), the middle group had 41 subjects (20 males and 21 females), and the old group had 58 subjects (23 males and 35 females). There was no statistically significant difference between the proportion of men and women in each group.

**Figure 6:**
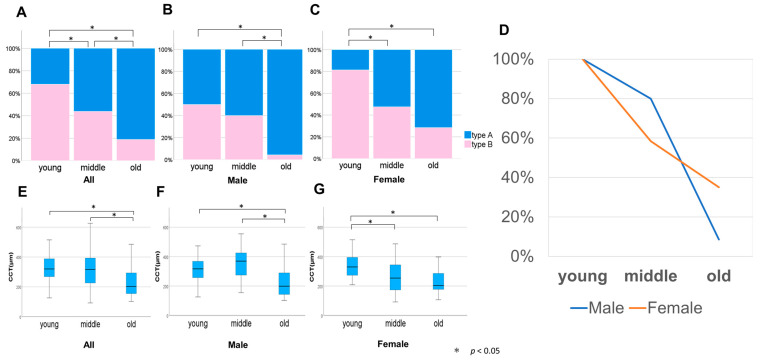
Types A and B in each age group. The proportions of groups A and B were compared by dividing them into three groups according to age: young, <40 years; middle, 40–60 years; old, ≥60 years. The proportions are as follows: (**A**) total, (**B**) male, and (**C**) female. (**D**) Line graph of the rate of decrease in group B, assuming that the ratio of group B in the young age group is 100% following normalization. Box-and-whisker plot of average central choroidal thickness for the three groups: (**E**) total, (**F**) male, and (**G**) female.

Figure 6A shows the proportions of the overall patterns for each age group: 31.9% and 68.1%, 56.1% and 43.9%, and 81.0% and 19.0% for types A and B in the young, middle-aged, and old groups, respectively. There was a significant difference in the proportions between the young and middle, young and old, and middle and old age groups (*p* < 0.05).

The pattern proportions for each age group for men are shown in Figure 6B. The results show that 50.0% and 50.0%, 60.0% and 40.0%, and 95.7% and 4.3% of types A and B were in the young, middle-aged, and old groups, respectively. There was no significant difference in the proportions between young and middle age; however, a significant difference was observed between the old and young (*p* < 0.05) and middle-aged groups (*p* < 0.05).

The pattern proportions of each age group for women are shown in Figure 6C. We observed that 18.5% and 81.5%, 52.4% and 47.6%, and 71.4% and 28.6% of types A and B in the young, middle-aged, and old groups, respectively. No significant difference in the proportions was observed between old and middle age; however, there was a significant difference between the young and middle-aged (*p* < 0.05) and old age groups (*p* < 0.05).

The change in the proportion of type B with age was demonstrated by calculating the ratio of type B at a young age as 100% following normalization. The proportion at middle age slightly decreased to 80.0% in men, while that in women decreased remarkably to 58.4%. However, the proportion at older age notably decreased to 8.6% in men, while that in women slightly decreased to 35.1% (Figure 6D). 

Since there was a different age-dependent change of choroidal thickness pattern between the sexes, CCT was analyzed (Figure 6E–G). The mean CCT in all the participants was 327.5 ± 84.4, 322.7 ± 133.1, and 231.3 ± 96.5 µm for those aged <40, 40–60, ≥60 years, respectively (Figure 6E). There was a significant difference between young versus old age (*p* < 0.05) and middle versus old age (*p* < 0.05).

The mean CCT in men was 311.6 ± 87.8, 359.9 ± 104.4, and 227.1 ± 102.3 µm for those <40, 40–60, and ≥60 years, respectively (Figure 6F). There was a significant difference between young versus old age (*p* < 0.05) and middle versus old age (*p* < 0.05).

In contrast, the mean CCT in women was 339.3 ± 81.4, 287.2 ± 149.6, and 231.3 ± 93.9 µm for those <40, 40–60, and ≥60 years, respectively (Figure 6G). There was a significant difference between young versus middle age (*p* < 0.05) and young versus old age (*p* < 0.05). The combinations that were statistically significant demonstrated a similar trend for the choroidal thickness maps and CCT.

## 4. Discussion

To the best of our knowledge, this is the first study to report the measurement of wide-area choroidal thickness from the posterior pole of the fundus to the vortex vein in healthy eyes, as well as the creation of a thickness map and pattern classification. The choroidal thickness map of healthy eyes based on the B-scan images allowed us to accurately characterize the structure of the choroid in three dimensions. Furthermore, the extensive information on choroidal thickness in healthy eyes could be captured more clearly by WF-OCT owing to the color display of its features.

We classified the choroidal maps based on the thickness of the choroid, especially above and below the optic disc. The presence of choroidal thickness greater than 250 µm above or below the optic disc, as in type B, indicates the presence of choroidal vessels crossing the watershed. This study demonstrated a significant correlation between CCT and the type of choroidal thickness map.

**Figure 7:**
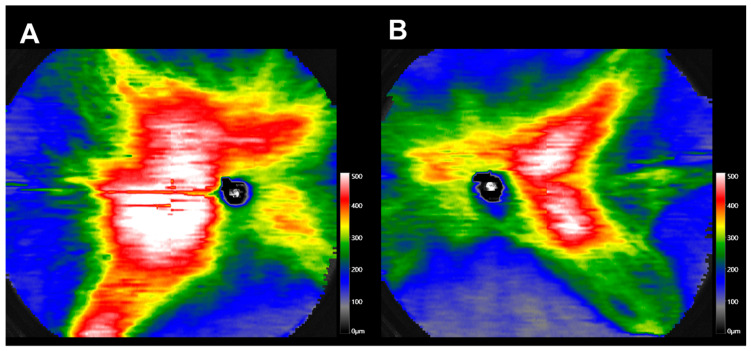
(**A**) A 37-year-old man with CSC. (**B**) A 41-year-old man with CSC.

In diseased eyes, such as PSD, CCT is a significant biomarker related to disease activity [18]. We observed several patients with PSD with type B2 pattern (a representative case is shown in Figure 7). Thus, peripapillary choroidal thickness and its pattern may relate to PSD activity.

The proportion of choroidal type B patterns, in which the supratemporal and supranasal regions are contiguous, decreased with age, and the proportion of type B patterns was higher in women than in men. Several reports exist on the effects of sex on the choroid. Men reportedly have significantly thicker choroids than women [13,19], and reports exist on the sex differences in the lumen/interstitial ratio [13]. We examined the number of vortex vein ampullae from montage fundus images and reported that the mean number of vortex veins was significantly higher in men than in women, which was more pronounced in the superior quadrant [20]. The lower number of vortex veins in women could result in a higher blood return volume per each Haller vessel in women. Thus, each vortex vein could be more prone to stasis of the returning blood, resulting in the thickening of the choroid in that area and a higher proportion of type B patterns in women.

The proportion of choroidal type B patterns decreased with age. Interestingly, the timing of the dynamic change of the ratio of type A to type B varies between the sexes. The ratio changed significantly after 60 years of age in men. However, in women, it changed significantly after 40 years of age. Although the effect of sex and age on a WF choroidal map has not been reported, certain studies have investigated the effect of sex and age on CCT. Ding et al. [21] reported a significant correlation between the age and CCT only in participants older than 60 years. Meanwhile, Ruiz-Medrano et al. [22] reported a negative correlation between the age and CCT, with a large change in the CCT between the 21–40-year age group (313.9 μm) and the 41–60-year age group (264.6 μm).

Although the results for both the groups were conflicting, they indicated that changes in the CCT vary after 40 or 60 years of age. We observed a significant change in the CCT between the 41–60 and ≥60-year-old age groups in the men in this study, similar to that described by Ding et al. However, for women, we observed a considerable variation in the CCT between the group aged ≤40 and 41–60 years, similar to that reported by Ruiz-Medrano et al.

During menopause, in their 40s and 50s, women undergo major biological changes. Estrogen is thought to play a major role in these changes. Estrogen is an important regulator of lipid metabolism, bone metabolism, inflammation, and vascular homeostasis. In addition, the secretion of female hormones increases in the late teens, peaks in the late 20s and 30s, and gradually declines, with a sharp decrease from the late 40s through menopause [23]. The rapid decrease in estrogen during menopause is thought to be associated with accelerated atherosclerosis and decreased cerebral blood flow [24,25].

In ophthalmology, Kavroulaki et al. [26] reported significantly more choroidal blood flow in younger women (aged < 40 years) than in older postmenopausal women (>55 years). The involvement of estrogen, which has a dilatory effect on the blood vessels, is believed to account for these findings. Male hormones are also thought to be associated with the choroidal thickness. Çiloğlu et al. [27] have revealed that the CCT is thicker and total testosterone levels are significantly increased in patients with CSC. Dehydroepiandrosterone, a regulator of ocular blood flow, affects the production of nitric oxide, which could influence the autoregulation of choroidal blood flow owing to androgen receptors present in the choroid and retinal pigment epithelium [28]. Male hormones decrease more slowly than female hormones; thus, choroidal changes are also expected to decrease more slowly. 

These sex-hormonal influences involve blood vessels and blood flow and may affect the macular and peripheral areas. The region above the optic disc is called the “watershed” and is a region where large choroidal vessels are relatively sparse. Thus, vascularity changes are more visible in the region than in other regions, including the posterior pole, where the choroidal vessels are relatively abundant. As a result, the changes in the choroidal thickness in the region above the optic disc are more easily observed. Thus, the difference in the timing of the change in the ratio of type B reduction and the reduction between men and women could be due to the gradual decrease in androgen in men, whereas the estrogen levels significantly decrease in perimenopausal women.

The strength of this study is the use of WF-OCT to create a choroidal thickness map and quantitatively evaluate the choroidal thickness over a wide area from the posterior pole to the vortex vein. Scattered reports exist on the choroidal thickness maps [12]; however, all of these maps were smaller than that obtained by the current method. In addition, the number of cases was limited since the segmentation of the scleral border was done manually, and great effort was required for image analysis. In this study, we employed a large number of healthy eyes (146 eyes) and produced a 20 × 23-mm-wide choroidal thickness map, including the fovea and optic disc. Segmentation was performed automatically using built-in software supported by artificial intelligence, which made it highly objective and allowed for a large number of analyses, thereby accommodating a wide range of ages.

Our study has certain limitations. First, the image classification was conducted through subjective qualitative assessment. Second, the applicability of the results to other ethnic groups is not yet known since only individuals of Japanese origin were included in the study. Third, the peripheral choroidal thickness may be affected by vortex vein location and morphology, but we could not analyze the location of the vortex veins in this study. We previously succeeded in visualizing all of the vortex veins using an ultra-widefield fundus camera. The relationship between the location of vortex veins and the pattern of choroidal thickness should be investigated in the future. In this study, we focused on the generational changes in choroidal thickness and the difference between type A and type B, that is, the choroidal thickness over the optic disc. Whether other peripheral choroidal thicknesses, in other words, the subtypes classified in this study, are important biomarkers needs to be investigated in the future.

In conclusion, we developed a wide-range choroidal thickness map for healthy eyes and classified choroid thickness into two patterns and five subtypes based on the characteristics of the choroidal thickness map. We established that the patterns varied between the sexes and that the proportions of these patterns varied between the age groups. Considering that the choroid is composed of blood vessels, it could reflect the effects of systemic and therapeutic treatments more strongly than the retina, which is a neural tissue with greater structural plasticity; thus, our study could provide basic data for this purpose.

## Figures and Tables

**Table 1:** Participant demographics.

Characteristics	Total	Male	Female	*p* Value (M vs. F)
		(N = 63)	(N = 83)	
Sex	Male 63 (43.2%)			
Age years, mean (SD)	52.3 ± 19.8	51.14 ± 19.18	53.10 ± 20.34	0.588
Axial lengths mm, mean (SD)	23.80 ± 1.18	24.18 ± 1.12	23.52 ± 1.15	0.02
Spherical equivalent diopter mean (SD)	−1.13 ± 1.81	−1.13 ± 1.81	−1.32 ± 1.91	0.891
CCT mm, mean (SD)	287.9 ± 113.8	296.05 ± 112.15	281.73 ± 115.28	0.337

Only the axial lengths were significantly different between males and females, while the age, central choroidal thickness (CCT), and spherical equivalent were not significantly different. SD, standard deviation; M, male; F, female.

**Table 2:** Multiple regression analysis with variable selection using stepwise method.

Factor	Multiple Regression Analysis (Corrected R2 = 0.213)	
	β Standardized Regression Coefficient	*p*
Age (year)	−0.52	<0.01
Axial lengths (mm)	−0.29	<0.01
Spherical equivalent (diopter)	-	-

## Data Availability

Data is available upon reasonable request to the corresponding author.
